# Biomarkers Detecting the Activity of ANCA‐Associated Vasculitis: A Systematic Literature Review

**DOI:** 10.1155/ijne/3133057

**Published:** 2025-12-22

**Authors:** L. Baas, R. M. Krol, E. C. Hagen, J. Spierings, Y. K. O. Teng, C. A. Koelman, H. H. F. Remmelts

**Affiliations:** ^1^ Department of Internal Medicine, Meander Medical Center, 3813, TZ Amersfoort, the Netherlands, meandermc.nl; ^2^ Department of Nephrology, Meander Medical Center, 3813, TZ Amersfoort, the Netherlands, meandermc.nl; ^3^ Department of Rheumatology & Clinical Immunology, University Medical Center Utrecht, 3584, CX Utrecht, the Netherlands, umcutrecht.nl; ^4^ Department of Medical Nephrology, Leiden University Medical Center (LUMC), 2333, ZA Leiden, the Netherlands, lumc.nl; ^5^ Department of Medical Immunology, Meander Medical Center, 3813, TZ Amersfoort, the Netherlands, meandermc.nl

**Keywords:** ANCA-associated vasculitis, biomarkers, CD163, CD206, CD25, EGPA, GPA, MCP-1, MPA

## Abstract

**Introduction:**

Anti‐neutrophil cytoplasmic antibody (ANCA)–associated vasculitis (AAV) encompasses a group of rare systemic autoimmune diseases characterized by inflammation of small‐ and medium‐sized blood vessels. Despite the efficacy of immunosuppressive therapy in achieving disease remission, a significant proportion of patients experience relapses, underscoring the need for reliable biomarkers to monitor disease activity. This systematic literature review evaluates the potential of urinary and serum biomarkers (CD163, CD206, CD25, and MCP‐1) to detect active AAV in adult patients.

**Method:**

A comprehensive search on PubMed, Embase, and Cochrane databases identified relevant studies, which were screened and assessed for inclusion based on predefined criteria. Data extraction and quality appraisal were independently conducted using the Quality Assessment Tool for Diagnostic Accuracy Studies (QUADAS‐2).

**Results:**

A total of 20 studies evaluated biomarkers for their diagnostic accuracy in detecting AAV activity. Most articles were scored as moderate risk of bias, with low concerns regarding applicability. Urinary soluble CD163 shows promising diagnostic accuracy for active renal vasculitis, with sensitivity and specificity values ranging from 0.72 to 1 and 0.67 to 0.98, respectively. Serum soluble CD163 and CD206 demonstrated variable accuracy. Serum MCP‐1 did not differ between patients in remission and patients with active disease, while urinary MCP‐1 showed potential but with inconsistent results across studies. Serum soluble CD25 was significantly elevated in active disease. Some combinations of biomarkers improved diagnostic performance (usCD163 + usCD25 + ssCD25 and usCD163 + serum Calprotectin + hematuria).

**Conclusion:**

In conclusion, while usCD163 individually appears to be the most reliable single biomarker for detecting active renal vasculitis in these studies, the heterogeneity of study designs and cutoff values across studies precludes definitive conclusions. Further research is necessary to standardize biomarker use, evaluate promising biomarker combinations, and improve the accuracy of activity monitoring both in renal and extrarenal AAV.

**Summary**

• **Identification of urinary soluble CD163 as a promising biomarker**: This review underscores the potential of urinary soluble CD163 (usCD163) as a reliable biomarker for detecting active renal vasculitis in AAV, with high sensitivity and specificity.
• **Exploration of biomarker combinations:** The study highlights innovative biomarker combinations (e.g., usCD163 + usCD25 + ssCD25, and usCD163 + serum Calprotectin + hematuria) that enhance the ability of detecting and more effective monitoring AAV activity.
• **Call for further research:** The findings emphasize the need for further research to standardize biomarker thresholds and outcome measures. Besides that, future research should focus on biomarkers not yet sufficiently investigated and promising combination of biomarkers
• **Toward personalized treatment with biomarkers:** Standardized, longitudinal evaluation of biomarkers across large cohorts can provide critical insights into their role throughout the AAV disease course. Detecting biomarkers that signal relapse risk will empower physicians to personalize the duration and intensity of maintenance treatments, optimizing patient outcomes.

## 1. Introduction

Anti‐neutrophil cytoplasmic antibody (ANCA)–associated vasculitis (AAV) is a group of relatively rare systemic autoimmune diseases, characterized by pauci‐immune necrotizing inflammation of the small‐ and medium‐sized blood vessels. AAV is typically associated with immune reactivity against two antigenic targets: proteinase 3 (PR3‐ANCA) and myeloperoxidase (MPO‐ANCA) [1, 2]. There are three subtypes of AAV: granulomatosis with polyangiitis (GPA), microscopic polyangiitis (MPA) and eosinophilic GPA (EGPA). There is a broad clinical spectrum of AAV, including involvement of the upper and lower respiratory tracts, kidneys, skin, brain, peripheral nerves, eyes and ear, nose, and throat (ENT) [3, 4].

AAV is a chronic relapsing and remitting disease. The cornerstone of treatment in AAV is immunosuppressive therapy. Most of the AAV patients achieve disease remission after treatment; however, between 20% and 56% of patients experience at least one relapse, mostly within 5 years after onset of the AAV [5–7]. Relapses are associated with increased risk for morbidity and mortality [8].

At this time, disease activity is being monitored by symptoms and markers such as C‐reactive protein and ANCA titer, but there is no reliable activity marker available [9]. The utility of serial ANCA measurements among patients with AAV to assess disease activity or predict disease relapse remains highly controversial. The published data on serial ANCA testing is heterogeneous. A meta‐analysis of Tomasson et al. calculated an estimated sensitivity and specificity of a rise in ANCA as a predictor of relapse of 0.56 (95% CI 0.33, 0.79) and 0.82 (95% CI 0.75, 0.90). They concluded that a rise in ANCA titer only modestly predicts subsequent relapse of disease and that routine ANCA testing does not alter clinical risk estimates enough to substantially affect clinical practice [2].

The pathophysiology of AAV remains insufficiently understood, but neutrophil activation and infection play an important role. Following inflammatory stimuli, neutrophils move intracellular ANCA antigens (MPO and PR3) to their surface, where autoantibodies bind and activate neutrophils [10].

More recently, the role of other cell types like macrophages has become more evident. Monocytes/macrophages enhance an inflammatory response, eliminate invading pathogens, and promote tissue repair [11]. Although ANCA antigens are key in AAV, ANCA negative AAV cases suggest other ANCA‐independent immune processes, such as T‐cell activation [10].

Emerging literature describes that CD163, CD206, CD25, and MCP‐1 are elevated at the time of a relapse and therefore could function as biomarkers in detecting a relapse or residual activity of AAV [11–13]. For example, a recent review of Renson et al. described that urinary levels of sCD163 are increased in active ANCA‐associated glomerulonephritis versus remission and that serum and urinary sCD25 can complement urinary sCD163 [14].

The biomarkers can be measured in both urine and serum, besides CD206, which is only measurable in serum. CD163 is the hemoglobin scavenger receptor expressed by macrophages and its expression is increased during inflammatory responses [15]. CD25, known as the IL‐2 receptor, is found on T cells and is involved in regulating cell proliferation and differentiation and has shown to have greater expression on CD4+ T cells during active disease in AAV compared with healthy controls [12, 16]. The mannose receptor CD206 is expressed by macrophages and plays a role in immune homeostasis [17]. MCP‐1 (also called chemoattractant CCL2), is a chemokine produced by nonhematopoietic cells (mainly monocytes/macrophages), promotes the expression of inflammatory mediators/cells and is a potent chemotactic factor for monocytes [18].

This study aimed to systematically review evidence for detecting active renal and extra‐renal AAV in adult patients with the following biomarkers: urinary and/or serum CD163, CD206, CD25, and MCP‐1.

## 2. Materials and Methods

### 2.1. Search Strategy and Selection Criteria

For this systematic literature review, a research question was formulated regarding biomarkers identifying AAV relapse/activity. The PICO‐method was used (Population, Intervention, Comparison, Outcome) with adult AAV patients as population; the four biomarkers as intervention/diagnostic tool; and AAV activity as outcome (Supporting Information S1).

A search string was developed including synonyms for the population and biomarkers (Supporting Table S1). The databases PubMed, Embase, and Cochrane were searched (august 2023).

Studies with adult AAV patients (minimum age of 18 years, according to the Chapel Hill Consensus Conference) evaluating diagnostic biomarkers were included. Exclusion criteria were: articles with no full‐text available articles not written in English, letters to editors, case reports, congress abstracts and animal studies. The references of articles were screened and relevant new articles not found in the search were added by the committee.

Two researchers screened all titles and abstracts of the remaining studies. After this, all remaining articles were screened in full‐text form. The screening was performed independently. Disagreements between the researchers were resolved through discussion.

### 2.2. Biomarkers and Outcome

Studies that assessed the usefulness of biomarkers (urinary and/or serum CD163, CD206, CD25, MCP‐1) in detecting a relapse or active AAV were included. Outcomes reflecting AAV activity/relapse were preferably described according to the Birmingham vasculitis activity score version 3 (BVAS‐3) [19].

### 2.3. Data Extraction and Critical Appraisal

The extraction of the data was performed independently by two authors for all articles. Information extracted from the articles included the name of the first author, year of publication, country, number of patients, type of study, type of AAV, activity score (also determination of active renal disease), biomarkers, and diagnostic test values.

The critical appraisal was performed using the Quality Assessment Tool for Diagnostic Accuracy Studies (QUADAS‐2), evaluating the risk of bias and applicability of the studies [20]. The QUADAS‐2 evaluates each study on four domains; patient selection, index test, reference standard, and timing. The four domains are critically appraised with the use of 14 questions (Table 1)*.* Studies were classified as low, moderate, or high risk of bias based on 14 questions across the four domains. All articles were assessed independently by two authors. Discrepancies were resolved through discussion.

**Table 1:** Quality assessment tool for diagnostic accuracy studies (QUADAS‐2).

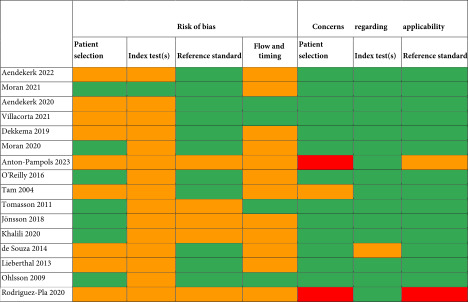

This review was conducted in accordance with the Preferred Reporting Items for Systematic Review and Meta‐Analysis (PRISMA) guidelines [21].

## 3. Results

**Figure 1:**
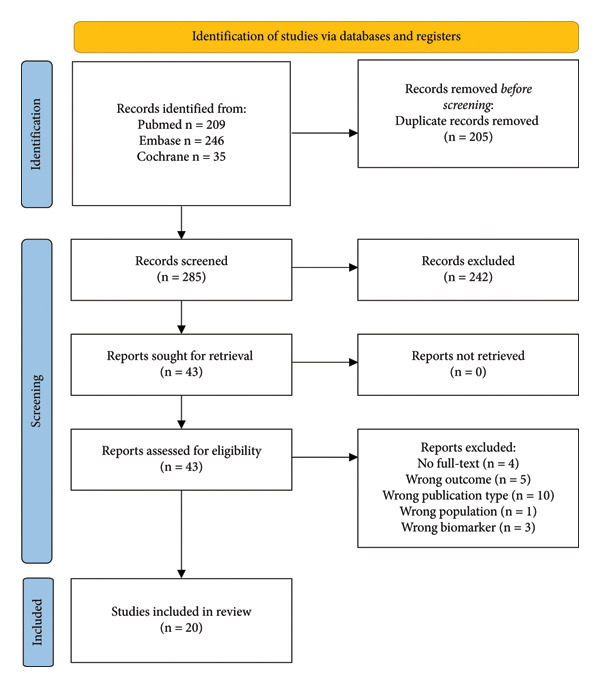
Flowchart of article selection.

A total of 490 records were retrieved from the search (Figure 1)*.* After the identification and removal of 205 duplicates, 285 records were screened on title and abstract. After this screening, 43 articles were assessed in full‐text and 20 articles were included, including a total of 1.949 AAV patients. Results of the included articles were presented per biomarker or a combination of biomarkers. The baseline characteristics of the studies are shown in Table 2, Table 3, Table 4, and Table 5. Each table represents a different (serum and/or urinary) biomarker or a combination of biomarkers.

**Table 2:** Overview of articles reporting on urinary soluble CD163, serum soluble CD163, and serum soluble CD206.

First Author country	Publication year	Study design	Biomarker	Number of AAV patients	AAV type	Activity score	Outcome	Active disease	Results	Risk of bias^∗^ concerns regarding applicability^∗^
Aendekerk [11] Netherlands, Ireland	2022	Retrospective cohort study	usCD163	210	AAV	BVAS3	Active ANCA GN	Kidney biopsy findings or new or increasing hematuria/proteinuria/rise in serum creatinine	Optimal cut off: 301.9 ng/mmol; AUC: 0.86; sensitivity: 0.72; specificity: 0.97	Moderate
										Low
Moran [25] Ireland	2021	Prospective cohort study	usCD163	84	AAV	BVAS3	Active ANCA GN	A combination of serum creatinine, hematuria, proteinuria and red blood cell casts	Optimal cut off: 250 ng/mmol; AUC: 0.95; sensitivity: 0.87; specificity: 0.97	Low
										Low
Aendekerk [24] Netherlands	2020	Retrospective cohort study	usCD163	95	AAV	Active ANCA GN in biopsy	Active ANCA GN	Active ANCA GN in biopsy	Optimal cut off: 30 ng/mmol AUC: 0.94 sensitivity: 0.94 specificity: 0.91	Low
										Low
Villacorta [27] Spain	2021	Prospective cohort study	usCD163	47	GPA, MPA, RLV	BVAS3	Active ANCA GN	Active urine sediment and/or an increase in creatinine concentration by > 30% attributable to active vasculitis	Optimal cut off: 20 ng/mmol absolute changes in usCD163 concentration; AUC: 0.97 sensitivity: 1; specificity: 0.88 optimal cut off: 20% relative changes in usCD163 concentration; AUC: 0.96; sensitivity: 1; specificity: 0.89	Low
										Low
Dekkema [12] Netherlands	2019	Retrospective cohort study	usCD163	154	AAV	BVAS3	Active ANCA GN	New or increasing hematuria/proteinuria, and/or in serum creatinine.	Optimal cut off: 350 ng/mmol; AUC: 0.91 sensitivity: 0.72; specificity: 0.98	Moderate
										Low
Moran [13] USA, Canada	2020	Retrospective cohort study	usCD163	88	AAV	BVAS	Active ANCA GN	New or worse hematuria/proteinuria/urinary RBC casts and/or an increase in serum creatinine > 30%	Optimal cut off: 72.9 ng/mmol; AUC: 0.79; sensitivity: 0.80; specificity: 0.67	Low
										Low
Anton‐Pampols [26] Spain	2023	Retrospective cohort study	usCD163	138	AAV	BVAS3	Active ANCA GN	New increase in hematuria/proteinuria/increase of ANCA and/or increase of creatinine serum along with/without new clinical symptoms.	Optimal cut off: 12 ng/mL; AUC: 0.88 sensitivity: 0.88; specificity: 0.80	Moderate
										Moderate
O′Reilly [22] Ireland	2016	Retrospective cohort study	usCD163	177	GPA, MPA, eGPA, anti‐GBM disease	BVAS3	Active ANCA GN	BVAS ≥ 0 with one or more renal items, including the presence of hematuria by urine dipstick	Optimal cut off: 0.3 ng/mmol; AUC: 0.93; sensitivity: 0.83; specificity: 0.96	Low
										Low
O′Reilly [22] Ireland	2016	Retrospective cohort study	ssCD163	177	GPA, MPA, EGPA, anti‐GBM disease	BVAS3	Active ANCA GN	BVAS ≥ 0 with one or more renal items, including the presence of hematuria by urine dipstick	No significant difference	Low
										Low
Dekkema [12] Netherlands	2019	Retrospective cohort study	ssCD163	154	AAV	BVAS3	Active ANCA GN	Clinical practice: New or increasing hematuria, and/or proteinuria, and/or in serum creatinine	Optimal cut off: 630 ng/mL; AUC: 0.66; sensitivity: 0.66; specificity: 0.72	Moderate
										Low
Aendekerk [11] Netherlands, Ireland	2022	Retrospective cohort study	ssCD206	210	AAV	BVAS3	Active ANCA GN	Kidney biopsy findings or new or increasing hematuria/proteinuria/rise in serum creatinine	Optimal cut off: 332.5 ng/mL; AUC: 0.72; sensitivity: 0.48; specificity: 0.87	Moderate
										Low

Abbreviations: AAV, ANCA‐associated vasculitis; ANCA GN, ANCA glomerulonephritis; anti‐GBM disease, anti glomerular basement membrane disease; AUC, area under the curve; BVAS, Birmingham Vasculitis Activity Score; eGPA, eosinophilic granulomatosis with polyangiitis; GPA, granulomatosis with polyangiitis; MPA, microscopic polyangiitis; RBC, red blood cell; RLV, renal limited vasculitis; ssCD163, serum soluble CD163; ssCD206, serum soluble CD206; usCD163, urinary soluble CD163.

^∗^The critical appraisal was performed using the Quality Assessment Tool for Diagnostic Accuracy Studies (QUADAS‐2), evaluating the risk of bias and applicability.

**Table 3:** Overview of articles reporting on serum MCP‐1 and urinary MCP‐1.

First author country	Publication year	Study design	Biomarker	Number of AAV patients	AAV type	Activity score	Outcome	Active disease	Results	Risk of bias^∗^ concerns regarding applicability^∗^
Tam [28] UK	2004	Prospective cohort study	sMCP‐1	52	AAV	BVAS	Active ANCA GN	Positive BVAS with renal involvement (mostly by kidney biopsy)	No significant difference	Moderate
										Low
Tomasson [29] USA	2011	Prospective cohort study	sMCP‐1	180	GPA	BVAS + PGA	Active GPA	BVAS > 0 after remission had been achieved	MCP‐1 was inversely associated with disease activity (OR 0.36)	Low
										Low
Moran [13] USA, Canada	2020	Retrospective cohort study	uMCP‐1	88	AAV	BVAS	Active ANCA GN	New or worse hematuria/proteinuria/urinary RBC casts and/or an increase in serum creatinine > 30%	Optimal cut off: 10 pg/mmol; AUC: 0.68; sensitivity: 0.54; specificity: 0.82	Low
										Low
Jönsson [32] Sweden	2018	Retrospective cohort study	uMCP‐1	113	GPA, MPA	BVAS	Active AAV	BVAS 2–5 = grumbling BVAS > 6 = relapse/active	The amount of MCP‐1 was significantly lower in patients in remission than in patients in the active phase (*p* = 0023)	Moderate
										Low
Khalili [23] Canada	2020	Prospective cohort study	uMCP‐1	18	AAV	BVAS	Active ANCA GN	Relapse is when a patient is not meeting a BVAS of 0 after it had been reached	Optimal cut off: 126 ng/mmol; AUC 0.78 (0.71–0.86)	Moderate
										Low
de Souza [31] Brasil, Netherlands	2014	Retrospective cohort study	uMCP‐1	24	GPA, MPA, RLV	BVAS3	Active ANCA GN	Glomerular erythrocyturia/decreased (eGFR) and/or with a renal biopsy showing active ANCA GN	AAV patients with active GN presented higher urinary MCP‐1 levels than HC (*P* = 0.035)	Moderate
										Low
Lieberthal [30] USA, Canada	2013	Prospective cohort study	uMCP‐1	50	AAV	BVAS + PGA	Active ANCA GN	Hematuria/RBC casts, and/or rise in serum creatinine > 30% that were interpreted by the clinician as being due to active ANCA GN	Optimal cut off: 1.3‐fold increase; AUC: 0.93 sensitivity: 0.94; specificity: 0.89	Moderate
										Low
Tam [28] UK	2004	Prospective cohort study	uMCP‐1	52	AAV	BVAS	Active ANCA GN	Positive BVAS with renal involvement (mostly by kidney biopsy)	uMCP‐1 levels were higher in patients with active (*P* < 0.01) or persistent ANCA GN (*P* < 0.05)	Moderate
										Low
Ohlsson [33] Sweden	2009	Retrospective cohort study	uMCP‐1	99	AAV	BVAS	Active AAV	BVAS < 5 = smoldering BVAS > 6 = relapse/new activity	uMCP‐1 levels correlates with severe outcome (*P* < 0.001) and there was a tendency toward higher levels in patients relapsing within 3 months (*P* = 0.08)	Low
										Low

Abbreviations: AAV, ANCA‐associated vasculitis; ANCA GN, ANCA glomerulonephritis; AUC, area under the curve; BVAS, Birmingham Vasculitis Activity Score; eGFR, estimated glomerular filtration rate; GPA, granulomatosis with polyangiitis; HC, healthy controls; MPA, microscopic polyangiitis; PGA, physician’s global assessment; RBC, red blood cell; RLV, renal limited vasculitis; sMCP‐1, serum MCP‐1; uMCP‐1, urinary MCP‐1.

^∗^The critical appraisal was performed using the Quality Assessment Tool for Diagnostic Accuracy Studies (QUADAS‐2), evaluating the risk of bias and applicability.

**Table 4:** Overview of articles reporting on serum soluble CD25 and urinary soluble CD25.

First author country	Publication year	Study design	Biomarker	Number of AAV patients	AAV type	Activity score	Outcome	Active disease	Results	Risk of bias^∗^ concerns regarding applicability^∗^
Aendekerk [24] Netherlands	2020	Retrospective cohort study	ssCD25	95	AAV	Active ANCA GN in biopt	Active ANCA GN	Active ANCA GN in biopsy	Optimal cut off: 1350 ng/L; AUC: 0.61; sensitivity: 0.35; specificity: 0.91	Low
										Low
Dekkema [12] Netherlands	2019	Retrospective cohort study	ssCD25	154	AAV	BVAS3	Active ANCA GN	New or increasing hematuria/proteinuria, and/or in serum creatinine.	Optimal cut off: 1050 pg/mL; AUC: 0.78; sensitivity: 0.62; specificity: 0.74	Moderate
										Low
Rodriguez‐Pla [35] USA	2020	Retrospective cohort study	ssCD25	37	eGPA	PGA	Active eGPA	PGA ≥ 2	IL‐2R showed significant increases in active disease (*P* = 0.01)	Moderate
										High
Schmitt [36] Germany	1998	Retrospective cohort study	ssCD25	16	eGPA	ELK classification + DEI	Active eGPA	Defined by a team of clinicians and extended imaging procedure. CR was defined as the absence of clinical, radiologic, and serologic evidence of disease activity.	sIL‐2R levels were significantly elevated during active eGPA (*P* < 0.005)	Moderate
										Low
Schmitt [37] Germany	1992	Retrospective cohort study	ssCD25	102	GPA	Extended ELK classification	Active GPA	Defined by an interdisciplinary team. CR was defined as the absence of clinical, radiologic, and serologic evidence of disease activity. PR was defined as improvement in disease manifestations or arrest of disease progression.	Levels of sIL‐2R correlate with disease activity in patients with GPA (*P* < 0.005).	Moderate
										Low
Sanders [39] Netherlands	2006	Retrospective cohort study	ssCD25	87	PR3 + AAV	BVAS	Active AAV	Clinical signs of vasculitic activity/the occurrence of nodular pulmonary lesions due to AAV, biopsy proved active AAV or glomerular hematuria/RBC casts/proteinuria and a decrease in creatinine clearance	At relapse, plasma sIL‐2R levels correlated significantly with BVAS (*p* < 0.01).	Low
										Low
Stegeman [38] Netherlands	1992	Retrospective cohort study	ssCD25	16	GPA	CRP and disease activity index/score	Active GPA	Minor relapses: Lesions of GPA in the upper or lower airways. Major relapses: Renal involvement (decreased renal function, RBC casts, biopsy proven active AAV), pulmonary involvement (respiratory failure), definite cerebral vasculitis or acute abdomen or massive gastrointestinal hemorrhage due to vasculitis	Levels of serum slL‐2R at a relapse correlated strongly with the disease activity scores at the moment of relapse (*P* < 0002).	Moderate
										Moderate
Aendekerk [24] Netherlands	2020	Retrospective cohort study	usCD25	95	AAV	Active ANCA GN in biopsy	Active ANCA GN	Active ANCA GN in biopsy	Optimal cut off: 210 ng/mmol; AUC: 0.50; sensitivity: 0.32; specificity: 0.82	Low
										Low
Dekkema [12] Netherlands	2019	Retrospective cohort study	usCD25	154	AAV	BVAS3	Active ANCA GN	New or increasing hematuria/proteinuria, and/or in serum creatinine.	Optimal cut off: 125 ng/mmol; AUC: 0.80; sensitivity: 0.67; specificity: 0.79	Moderate
										Low

Abbreviations: AAV, ANCA‐associated vasculitis; ANCA GN, ANCA glomerulonephritis; AUC, area under the curve; BVAS, Birmingham Vasculitis Activity Score; CR, complete remission; DEI, disease extension index; GPA, granulomatosis with polyangiitis; PGA, physician’s global assessment; PR, partial remission; PR3+, proteinase 3 positive; RBC, red blood cell; ssCD25, serum soluble CD25; usCD25, urinary soluble CD25.

^∗^The critical appraisal was performed using the Quality Assessment Tool for Diagnostic Accuracy Studies (QUADAS‐2), evaluating the risk of bias and applicability.

**Table 5:** Overview of articles reporting on a combination of biomarkers.

First author	Publication year	Study design	Biomarkers	Number of AAV patients	AAV type	Activity score	Outcome	Active disease	Results	Risk of bias^∗^ concerns regarding applicability^∗^
Aendekerk [11] Netherlands, Ireland	2022	Retrospective cohort study	usCD163 + ssCD206	210	AAV	BVAS3	Active ANCA GN	Kidney biopsy findings or new or increasing hematuria/proteinuria/rise in serum creatinine	Optimal cut off: see above; AUC: 0.83 sensitivity: 0.81; specificity: 0.84	Moderate
										Low
Aendekerk [24] Netherlands	2020	Retrospective cohort study	usCD163 + usCD25 + ssCD25	95	AAV	Active ANCA GN in biopsy	Active ANCA GN	Active ANCA GN in biopsy	Optimal cut off: see above; AUC: 0.92 sensitivity: 0.94; specificity: 0.91	Low
										Low
Dekkema [12] Netherlands	2019	Retrospective cohort study	usCD163 + usCD25 + ssCD25	154	AAV	BVAS3	Active ANCA GN	New or increasing hematuria/proteinuria, and/or in serum creatinine	Optimal cut off: see above; AUC: ‐Sensitivity: 0.85; specificity: 0.95	Moderate
										Low
Moran [13] USA, Canada	2020	Retrospective cohort study	usCD163 + uMCP‐1 + proteinuria	88	AAV	BVAS	Active ANCA GN	New or worse hematuria/proteinuria/urinary RBC casts and/or an increase in serum creatinine > 30%	Optimal cut off: usCD163 > 143 ng/mmol uMCP1 > 20 pg/mmol; new/worse proteinuria; AUC: ‐; sensitivity: 0.41; specificity: 0.98	Low
										Low
Anton‐Pampols [26] Spain	2023	Retrospective cohort study	usCD163 + sCalprotectin + hematuria	138	AAV	BVAS3	Active ANCA GN	New increase in hematuria/proteinuria/increase of ANCA and/or increase of creatinine serum along with/without new clinical symptoms.	Optimal cut off:usCD163 > 12 ng/mL; sCalprotectin > 5135 ng/mL; AUC: ‐; sensitivity: 0.97; specificity: 0.90	Moderate
										Moderate

Abbreviations: AAV, ANCA‐associated vasculitis; ANCA GN, ANCA glomerulonephritis; AUC, area under the curve; BVAS, Birmingham Vasculitis Activity Score; RBC, red blood cell; sCalprotectin, serum calprotectin; ssCD206, serum soluble CD206; ssCD25, serum soluble CD25; usCD163, urinary soluble CD163; usCD25, urinary soluble CD25.

^∗^The critical appraisal was performed using the Quality Assessment Tool for Diagnostic Accuracy Studies (QUADAS‐2), evaluating the risk of bias and applicability.

### 3.1. Study Characteristics

Studies were published from 1992 to 2023. Sample sizes varied between 16 and 210 AAV patients per study. AAV subtypes were GPA (*N* = 18 studies), MPA (*N* = 15 studies), and EGPA (*N* = 13 studies). Two studies included the subtype renal limited vasculitis (RLV), defined as the presence of isolated renal involvement, ANCA positivity, and/or biopsy‐proven pauci‐immune necrotizing glomerulonephritis. The study of O’Reilly et al. used the term small vessel vasculitis (SVV) and also included 11 patients (out of 465 patients in total) with anti–glomerular basement membrane disease (anti‐GBM) [22]. In all studies, Enzyme‐Linked Immunosorbent Assay (ELISA) was used to test the biomarkers (mostly from R&D Systems, Minneapolis, MN, USA) (see Supporting Table S2). A total of 9 out of 20 studies calculated and reported sensitivity, specificity, and area under the curve (AUC) of the biomarkers [11–13, 22–27], whereas the study of Khalili et al. only reported the AUC [28]. The rest of the studies mainly described whether there was a significant difference in biomarker concentration between patients with active vasculitis and patients in disease remission [29–38]. Most of the studies also compared biomarker levels of AAV patients with levels of biomarkers in healthy controls [11, 12, 22–25, 27, 29, 31, 32, 35–38].

### 3.2. Urinary Soluble CD163

Eight studies investigated the performance of urinary soluble CD163 (usCD163) (Table 2) in detecting active renal vasculitis. None of these studies investigated if usCD163 could detect extrarenal activity. Disease activity was determined using the BVAS‐3 and active kidney vasculitis was assessed according to clinical practice in most studies (active urinary sediment and/or rise in serum creatinine). Both studies of Aendekerk et al. used kidney biopsy findings as a gold standard to determine if there was active glomerulonephritis [11, 23]. The overall risk of bias was scored as moderate, except for the study of Moran et al., which was scored as having a low risk of bias [25]. Concerns regarding applicability were low for almost all studies, except for the study of Anton‐Pampols et al. where there were moderate concerns, because of concerns regarding applicability in patient selection (only new AAV patients were included, no relapsing patients) [24]. All studies found higher levels of usCD163 in patients experiencing a relapse, compared to those in remission or healthy controls (when included). The studies determined an optimal cutoff and calculated the sensitivity and specificity of usCD163 in predicting a renal relapse. Cutoff values ranged from 0.3 to 350 ng/mmol and were all corrected for urinary creatinine levels to correct for urinary dilution. Sensitivity ranged from 0.72 to 1 and specificity from 0.67 to 0.98. The study of Villacorta et al. used relative and absolute changes in usCD163 concentration (20%, respectively 20 ng/mmol) and found the highest sensitivity of 1.0, with a specificity of 0.89, respectively 0.8 [27].

### 3.3. Serum Soluble CD163

Two studies researched whether serum soluble CD163 (ssCD163) (Table 2) is able to detect active renal vasculitis. These studies did not explore whether ssCD163 is a marker for extrarenal AAV activity. Disease activity was scored with the BVAS‐3 in combination with clinical practice/renal items (new or increasing hematuria, and/or proteinuria and/or a rise in serum creatinine) in both studies. The overall risk of bias was scored as moderate and concerns regarding applicability were low. The study of O’Reilly et al. found no significant difference in ssCD163 concentration between patients with active renal vasculitis and patients in remission [22]. Dekkema et al. found an optimal cutoff of 630 ng/mL with a sensitivity of 0.66 and a specificity of 0.72 [12].

### 3.4. Serum Soluble CD206

Only one of the studies of Aendekerk et al. investigated if serum soluble CD206 (ssCD206) could detect an active renal vasculitis (Table 2)*.* This study did not research if ssCD206 could detect extrarenal activity. The overall risk of bias was scored as moderate and concerns regarding applicability were low. Disease activity was determined by the BVAS‐3 and active kidney vasculitis was assessed using kidney biopsy. When kidney biopsy was unavailable, activity was assessed according to clinical practice, defined as new or increasing erythrocyturia, and/or proteinuria, and/or a rise in serum creatinine. This study found higher levels of ssCD206 in relapsing patients, compared to those in remission of healthy controls. An optimal cutoff of 332.5 ng/mL was found, with a sensitivity of 0.48 and a specificity of 0.87 [11].

### 3.5. Serum MCP‐1

The studies of Tam et al. and Tomasson et al. explored whether serum MCP‐1 (sMCP‐1) is a marker of active vasculitis (Table 3)*.* The overall risk of bias was scored as moderate and concerns regarding applicability were low. Tam et al. investigated sMCP‐1 as a marker for active renal vasculitis, determined by positive BVAS‐3 with renal involvement. Tomasson et al. only included patients with GPA and investigated sMCP‐1 as a marker for an active GPA (renal and extrarenal), defined as a BVAS‐3 > 0 after remission had been achieved. Tam et al. found similar levels of MCP‐1 in the serum of patients with active renal vasculitis and remission. Tomasson et al. found that MCP‐1 was inversely associated with disease activity (OR 0.36, 95% confidence interval 0.22–0.57) [34, 38].

### 3.6. Urinary MCP‐1

Seven studies investigated the diagnostic accuracy of urinary MCP‐1 (uMCP‐1) (Table 2)*.* Most of the studies (*N* = 5) explored the ability of the biomarker to detect an active renal vasculitis, but the studies of Ohlsson et al. and Jöhnsson et al. looked at active AAV in general. The overall risk of bias was scored as moderate, except for the study of Ohlsson et al., which was scored as low risk of bias. Concerns regarding applicability were low. All studies defined disease activity using the BVAS‐3, with different definitions of active disease (see Table 4)*.* Active renal vasculitis was mostly assessed by the clinician according to clinical practice of renal involvement, mostly defined as new or increasing hematuria, and/or proteinuria and/or in serum creatinine or sometimes proven by kidney biopsy. Most studies found elevated levels of uMCP‐1 in active renal disease, when compared to patients in remission or healthy controls.

Two studies calculated optimal cutoff values, AUC, and sensitivity/specificity, and one study calculated only the optimal cutoff and AUC. Moran et al. found an optimal cutoff of 10 pg/mmol, with a sensitivity of 0.54 and a specificity of 0.82 [13]. Lieberthal et al. chose the optimal cutoff of 1.3‐fold increase of uMCP‐1, with a sensitivity of 0.94 and a specificity of 0.89 [26]. Khalili et al. found an optimal cutoff of 126 ng/mmol with an AUC of 0.78 [28]. The other four studies only investigated whether uMCP‐1 values were significantly higher in samples of patients with active disease compared to patients in remission. Three studies found significantly higher levels of uMCP‐1 in patients with active renal disease [32, 35, 38]. Ohlsson et al. described a tendency toward higher levels in patients relapsing within 3 months (general AAV relapse), however, not reaching statistical significance [36].

### 3.7. Serum Soluble CD25

Seven studies evaluated whether serum soluble CD25 (ssCD25) could detect active vasculitis (Table 4)*.* Aendekerk et al. and Dekkema et al. investigated the ability of the biomarker to predict an active renal vasculitis, while the other studies looked at a disease activity of AAV in general. The overall risk of bias was scored as moderate and concerns regarding applicability were low, except for the study of Rodrigues‐Pla et al., in which there were moderate concerns. Dekkema et al. defined active renal disease by clinical practice, and Aendekerk et al. by active AAV in kidney biopsy. They defined optimal cutoffs and sensitivity/specificity for detecting active renal disease. Aendekerk et al. found a cutoff of 1,350 nL/L, with a sensitivity of 0.35 and a specificity of 0.91 [23]. The optimal cutoff found by Dekkema et al. was 1,050 pg/mL with a sensitivity of 0.62 and a specificity of 0.74 [12]. Rodriguez‐Pla et al. and Schmitt et al. looked at eGPA, whereas Stegeman et al. and another study of Schmitt et al. looked at GPA and Sanders et al. only at PR3‐positive AAV. Disease activity was assessed differently by the studies, varying from using the BVAS‐3 to the ELK classification [classification based on anatomic site of involvement: upper airway or ENT (designated E), lung (L), and kidney (K)] [39], CRP and disease activity index/score and the physician’s global assessment of disease activity. These five studies only investigated if ssCD25 levels were significantly elevated in active disease. All studies found a significant correlation between serum levels and active disease, when compared to patients in remission or healthy controls (when in included) [29–31, 33, 37].

### 3.8. Urinary Soluble CD25

Aendekerk et al. and Dekkema et al. investigated whether urinary soluble CD25 (usCD25) is able to detect active renal vasculitis (Table 4). This study did not research if usCD25 could detect extrarenal activity. The overall risk of bias was scored as moderate, and concerns regarding applicability were low. They calculated optimal cutoff values and sensitivity/specificity. Aendekerk et al. found an optimal cutoff of 210 ng/mmol with a sensitivity of 0.32 and a specificity of 0.82 [23]. The cutoff calculated by Dekkema et al. was 125 ng/mmol with a sensitivity of 0.67 and a specificity of 0.79 [12].

### 3.9. Combination of Biomarkers

Five studies investigated whether a combination of different biomarkers or combining biomarkers with traditional markers could identify a renal flare in AAV (Table 5)*.* None of the studies investigated whether a combination of biomarkers could identify an extrarenal relapse. Both studies of Aendekerk et al. combined usCD163 with other biomarkers. The first study looked at a combination of usCD163 and ssCD206 with the use of the above‐mentioned cutoff values. They found a sensitivity of 0.81 and a specificity of 0.84. The combination increased sensitivity (from 0.72 to 0.81), however at the cost of specificity (from 0.97 to 0.84) [11]. The other study of Aendekerk et al. and Dekkema et al. investigated the combination of usCD163, usCD25, and ssCD25, and used the above‐mentioned cutoff values. A sensitivity of 0.94 and a specificity of 0.91 were found by Aendekerk et al. equivalent to the performance of usCD163 alone. Dekkema et al. found a sensitivity of 0.85 and a specificity of 0.95, showing an increase in sensitivity compared to usCD163 alone [12, 23]. Moran et al. evaluated the combination of CD163, uMCP‐1, and presence of proteinuria. The optimal cutoffs were: for usCD163 > 143 ng/mmol, for uMCP1 > 20 pg/mmol, and new/worsening proteinuria. They calculated a sensitivity of 0.41 and a specificity of 0.98. The combination increased specificity, however at the cost of sensitivity [13]. The last study investigated the combination of biomarkers usCD163, serum calprotectin (sCalprotectin), and hematuria. An optimal cutoff for usCD163 of 12 ng/mL and for sCalprotectin of 5135 ng/mL was used. The calculated sensitivity was 0.97 and the specificity 0.90, showing an increase of both the sensitivity and specificity of usCD163 alone [24].

## 4. Discussion

This systematic review aimed to evaluate the evidence on urinary and serum biomarkers, specifically CD163, CD206, CD25, and MCP‐1, and combinations thereof for detecting disease activity in AAV. These biomarkers are products of macrophages (CD163/CD206), lymphocytes (CD25), and various nonhematopoietic cells (MCP‐1), which all play an important role in the pathophysiology of AAV [11–13]. Results were presented per biomarker (Table 2, Table 3, Table 4, and Table 5). Most studies (12 out of 20) were scored as moderate risk of bias, with low concerns regarding applicability (except three studies) (Table 1).

Among these biomarkers, usCD163 consistently demonstrates the highest diagnostic accuracy for detecting active renal vasculitis [11–13, 22–25, 27]. Compared to the other biomarkers, usCD163 showed the highest sensitivity and specificity overall. Six of the eight studies also assessed the positive predictive value (PPV) and negative predictive value (NPV) of usCD163 as a diagnostic tool [11–13, 22–25, 27]. While the results vary slightly across studies, the overall predictive values are promising, with both PPV and NPV typically exceeding 0.8 on average. The PPV and NPV of a biomarker are critical for clinical decision‐making, especially in the context of monitoring ANCA vasculitis. A high NPV is particularly valuable, as it provides physicians with confidence that they are not missing active disease in their patients. This ensures that clinicians can avoid unnecessary treatments or interventions in patients who do not have a relapse. On the other hand, a high PPV enhances a doctor’s ability to more accurately assess when the disease is active, potentially identifying patients who may require earlier or intensified treatment. In this way, biomarkers with strong PPV and NPV can significantly improve clinical outcomes by aiding in timely and appropriate therapeutic decisions, reducing the risk of both undertreatment and overtreatment. The limited number of studies on ssCD163 and mixed results suggest that ssCD163 might be less reliable than its urinary counterpart, but further research is needed [12, 22]. During active AAV, CD163 is actively secreted into the urine by activated macrophages which are infiltrated in glomerular and interstitial compartments [40]. Due to the high molecular weight of CD163, this biomarker appears in the urine primarily when the integrity of the GBM is compromised by inflammation [11, 27]. This pathophysiological link further strengthens the reliability of usCD163 as a specific marker for renal flares in AAV [11, 12]. This is in contrast to sCD163, which is primarily cleared by the liver and is influenced by systemic inflammatory processes that may not directly reflect vasculitic activity [41].

Besides usCD163, the biomarker uMCP‐1 also demonstrated promise in predicting active vasculitis, however with varying degrees of diagnostic accuracy across the seven included studies [13, 26, 28, 32, 35, 36, 38]. Five studies found an association of elevated levels in active renal disease, although results varied widely, especially regarding sensitivity and specificity with a lowest reported sensitivity of 0.54 [13, 26, 28, 32, 38]. The optimal cutoff values varied widely, further complicating the interpretation of results. Two studies researched the detection of general AAV activity. One reported lower uMCP‐1 levels in patients in remission [35]. The studies on sMCP‐1 produced negative results, with one study finding no significant difference in MCP‐1 levels between active and remission states, while another found an inverse relationship with disease activity [34, 38]. Similar to usCD163, uMCP‐1 seems to be primarily associated with renal disease activity, likely due to the strong presence of MCP‐1 in inflamed renal tissue and the local production of MCP‐1 by kidney cells recruiting macrophages [13, 38]. Besides the role of MCP‐1 in inflammatory cell recruitment, MCP‐1 is also involved in tissue repair and can directly start a fibrotic response in glomerular mesangial cells [18, 42, 43]. This may explain the variation in the studies, since persistent elevation of uMCP‐1 during clinical remission could indicate ongoing repair processes, fibrotic remolding, chronic kidney damage, or subclinical renal inflammation [35, 36, 44].

The study of Ohlsson et al. specifically investigated uMCP‐1 as a predictor of future flares [36]. They found a tendency toward higher levels in patients relapsing within 3 months, however not statistically significant. These results are currently insufficient to support this biomarker as a reliable prognostic tool. Future research should ideally explore more biomarkers’ prognostic value, as this could be clinically valuable for early detection of impending flares or the risk of developing kidney damage.

Studies consistently found elevated ssCD25 levels in active disease [12, 23, 29–31, 33, 37], but only two studies, which specifically researched renal activity, reported sensitivity and specificity. The rest only studied whether there is a correlation between the level of ssCD25 and general AAV disease activity. Three of the studies evaluating ssCD25 used outdated disease activity scores, impairing comparison to other studies and current clinical practice [29, 30, 37]. Similar to ssCD25, usCD25 showed potential in two studies detecting active renal vasculitis, but with variability in cutoff values and diagnostic accuracy [12, 22]. The elevation of CD25 during relapses reflects T‐cell activation as CD25 is shed from activated T‐cells during inflammatory responses, reflecting T‐cell activation during AAV relapse. CD25 seems to be elevated in more inflammatory conditions, such as infections and hematological malignancies [29–31, 37, 45]. The literature also suggests incomplete suppression of T‐cell activation despite immunosuppressive treatment in AAV [29, 30]. The observation that sCD25 levels are higher in active glomerulonephritis may reflect increased immune activation within the renal microenvironment and reduced renal clearance plus tubular reabsorption of this medium‐sized protein during renal inflammation, leading to systemic accumulation [46]. In conclusion, urine and serum CD25 may be associated with disease activity, its diagnostic performance and applicability however may not be robust enough yet for clinical use without additional markers.

The mannose receptor CD206 has received limited attention in AAV biomarker research up to now. CD206 is only measurable in serum, because of its large size. CD206 is not freely filtered by the glomerulus, and there is no evidence of local production or shedding of CD206 within the renal parenchyma [11]. Only one study investigated ssCD206, finding a sensitivity of 0.48 and specificity of 0.87 in detecting ANCA glomerulonephritis [11]. The particularly strong elevation in renal relapse suggests tissue‐specific macrophage activation, consistent with CD206 localization to renal tubuli and sensitivity to tubulointerstitial inflammation [11, 47].

Five studies explored the diagnostic accuracy of combinations of biomarkers or biomarkers in combination with regular markers in detecting renal activity [11–13, 23, 24]. These combinations generally improved sensitivity, but sometimes at the cost of specificity. In one study, the combination of usCD163, usCD25, and ssCD25 showed an increase in sensitivity compared to usCD163 alone. A combination of usCD163, sCalprotectin and hematuria showed an increase of both the sensitivity and specificity compared to usCD163 alone. Combinations of biomarkers or combining them with traditional markers which we use nowadays may balance the strengths and weaknesses of individual biomarkers, leading to more reliable predictions of disease activity.

The primary strength of this review is its comprehensive scope, encompassing a broad range of studies over three decades. This inclusivity allows for a detailed examination of the biomarkers in question. By grouping the studies according to the specific biomarker investigated, the review offers a clear and organized overview of the available evidence, which aids in understanding the potential diagnostic value of each biomarker. Additionally, the use of standardized assessment tools, such as the QUADAS‐2 for evaluating the quality of the included studies, adds rigor to the review process. This methodological approach ensures that the findings are based on high‐quality evidence and provides a clear picture of the current state of research on biomarkers in AAV.

Despite its strengths, the review also highlights significant limitations within the existing body of research. One major challenge is the variability in cutoff values and outcome measures across studies. This inconsistency makes direct comparison difficult and precludes the possibility of performing a meta‐analysis and or to make clinical recommendations. Another limitation is the heterogeneity in study designs and patient populations. A significant finding from this review is the limited information on biomarkers for nonrenal disease activity in AAV, because most studies focused specifically on renal involvement. This variability can influence the sensitivity and specificity of the biomarkers, as different disease manifestations may alter biomarker levels differently. For example, the most promising biomarkers usCD163 and uMCP‐1 have mainly been studied concerning renal involvement. Therefore, it remains unclear whether these markers are useful for detecting AAV activity outside the kidneys. Only a few studies included in this review explored biomarkers for systemic or extra‐renal activity, with limited results. Consequently, reliable conclusions cannot be drawn about the diagnostic value of biomarkers for nonrenal manifestations of AAV based on this overview. The lack of data on specific extra‐renal involvement indicates a knowledge gap and provides an opportunity for studies to further evaluate these biomarkers in broader AAV manifestations. Besides that, none of the studies corrected the urinary biomarkers for the biomarker in the serum. The question is whether a biomarker in urine without correction for serum is a good measure of inflammation/activity of disease. Perhaps the biomarkers should always be measured simultaneously in both serum and urine to obtain urine/serum ratios as a reliable biomarker for disease activity.

To advance the field, future research should focus on standardizing cutoff values and outcome measures, biomarkers not yet sufficiently investigated and promising combination of biomarkers. Large‐scale studies with well‐defined patient populations and standardized protocols are essential to find out if the urinary and serum biomarkers can detect extra‐renal disease activity as well and if changes over time in urine, serum or urine/serum ratios can detect disease activity. Standardized longitudinal evaluation of the biomarkers in a large cohort is needed to give insight into the change of biomarkers throughout the disease course, from active generalized disease and induction treatment, toward remission and maintenance therapy. If patients at risk for a relapse can be detected, physicians will be enabled to personalize duration and intensity of maintenance treatment.

In conclusion, this systematic review highlights that there is significant variability in cutoff values and outcome measures for any of the potential of urinary and serum biomarkers in detecting AAV activity. UsCD163 emerges as a promising biomarker for detecting active renal vasculitis (whether or not in combination with other biomarkers). However, the variability in existing studies underscores the need for standardized, high‐quality research to validate these findings as well as to validate how these biomarkers can impact daily clinical practice. Notably, the value of these biomarkers helps to detect nonrenal disease activity in AAV patients.

**Nomenclature**
ANCA: anti‐neutrophil cytoplasmic antibody
AAV: anti‐neutrophil cytoplasmic antibody associated vasculitis
PR3‐ANCA: proteinase 3
MPO‐ANCA: myeloperoxidase
GPA: granulomatosis with polyangiitis
MPA: microscopic polyangiitis
eGPA: eosinophilic granulomatosis with polyangiitis
ENT: ear, nose, and throat
sIL‐2R: soluble IL‐2 receptor
PICO: Population, Intervention, Comparison, Outcome
BVAS‐3: Birmingham vasculitis activity score version 3
QUADAS‐2: Quality Assessment Tool for Diagnostic Accuracy Studies
PRISMA: Preferred Reporting Items for Systematic Review and Meta‐Analysis
SVV: small vessel vasculitis
anti‐GBM: anti‐glomerular basement membrane disease
ELIS: Enzyme‐Linked Immunosorbent Assay
AUC: area under the curve
RLV: renal limited vasculitis
usCD163: urinary soluble CD163
ssCD163: serum soluble CD163
ssCD206: serum soluble CD206
sMCP‐1: serum MCP‐1
uMCP‐1: urinary MCP‐1
ssCD25: serum soluble CD25
ELK: classification based on anatomic site of involvement: upper airway or ear, nose, and throat (designated E), lung (L), and kidney (K)
usCD25: urinary soluble CD25
sCalprotectin: serum calprotectin
ANCA GN: ANCA glomerulonephritis
RBC: red blood cell
HC: healthy controls
PGA: physician’s global assessment
CR: complete remission
DEI: disease extension index
PR: partial remission.

## Ethics Statement

The authors have nothing to report.

## Consent

The authors have nothing to report.

## Conflicts of Interest

The authors declare no conflicts of interest.

## Funding

The authors received no specific funding for this work.

## Supporting Information

Supporting Table S1 provides the PICO‐formulated research question and the full search strategies used for PubMed, Embase, and Cochrane.

Supporting Material S1 includes the complete search strings as applied in each database.

Supporting Table S2 lists the biomarker assay kits and suppliers used for the measurement of CD163, CD206, CD25, and MCP‐1.

## Supporting information


**Supporting Information** The supporting material accompanying this manuscript contains additional methodological details and supporting data.

## Data Availability

Data sharing not applicable to this article as no datasets were generated or analysed during the current study.
