# Supplementary material for: Biomarkers Detecting the Activity of ANCA‐Associated Vasculitis: A Systematic Literature Review
Source: Int J Nephrol. 2025 Dec 22;2025:3133057. doi: 10.1155/ijne/3133057 (PMC12752823; doi:10.1155/ijne/3133057)
Supplement: Supplementary file 1 — Supporting Information The supporting material accompanying this manuscript contains additional methodological details and supporting data. [file IJNE-2025-3133057-s001.docx]

**Supplementary Table S1**

*PICO formulated question*

| **Question:** What is the ability of the biomarkers CD163, CD206, CD25 and MCP-1 to detect an active AAV? |
| --- |
| Population: Adult patients with AAV  Intervention (diagnostic tool): biomarkers (CD163, CD206, CD25, MCP-1)  Comparison: usual care/BVAS  Outcome: AAV activity |

**Supplementary Material S1***Search string*

Pubmed
A search with this search string was performed on 02-08-2023

(((((((((((((((((((((((((((((((((((((((((((((((((anti-neutrophil cytoplasmic autoantibody associated vasculit*[Title/Abstract]) OR (Anti-Neutrophil Cytoplasmic Antibody-Associated Vasculit*[Title/Abstract])) OR (Anti Neutrophil Cytoplasmic Antibody Associated Vasculit*[Title/Abstract])) OR (ANCA-vasculit*[Title/Abstract])) OR (ANCA-Associated Vasculit*[Title/Abstract])) OR (ANCA Associated Vasculit*[Title/Abstract])) OR (GPA[Title/Abstract])) OR (EGPA[Title/Abstract])) OR (MPA[Title/Abstract])) OR (granulomatosis with polyangiitis[Title/Abstract])) OR (eosinophilic granulomatosis with polyangiitis[Title/Abstract])) OR (microscopic polyangiitis[Title/Abstract])) OR (Churg Strauss[Title/Abstract])) OR (Churg-Strauss[Title/Abstract])) OR (Eosinophilic Granulomatous Vasculit*[Title/Abstract])) OR (Wegener*[Title/Abstract])) OR (Microscopic Polyangiitides[Title/Abstract]) ) AND (CD163*[Title/Abstract])) ) OR (sCD163*[Title/Abstract])) OR (usCD163*[Title/Abstract])) OR (CD25*[Title/Abstract])) OR (sCD25*[Title/Abstract])) OR (usCD25*[Title/Abstract])) OR (Interleukin 2 Receptor*[Title/Abstract])) OR (Interleukin-2 Receptor*[Title/Abstract])) OR (Interleukin-2R*[Title/Abstract])) OR (Interleukin 2R*[Title/Abstract])) OR (IL-2R*[Title/Abstract])) OR (IL 2R*[Title/Abstract])) OR (CD206*[Title/Abstract])) OR (sCD206*[Title/Abstract])) OR (usCD206*[Title/Abstract])) OR (Mannose receptor[Title/Abstract])) OR (Mannose Receptor 1[Title/Abstract])) OR (Mannose Receptor C-Type I[Title/Abstract])) OR (MRC1 protein[Title/Abstract])) OR (sMCP-1[Title/Abstract])) OR (CCL2[Title/Abstract])) OR (Monocyte Chemotactic[Title/Abstract] AND Activating Factor[Title/Abstract])) OR (Monocyte Chemoattractant Protein-1[Title/Abstract])) OR (Monocyte Chemoattractant Protein 1[Title/Abstract])) OR (Chemokine (C-C Motif) Ligand 2[Title/Abstract])) OR (Monocyte Chemotactic Protein-1[Title/Abstract])) OR (Monocyte Chemotactic Protein 1[Title/Abstract])) OR (MCP-1[Title/Abstract])) OR (MCP 1[Title/Abstract])) OR (biomarker[Title/Abstract])) OR (Diagnostic marker[Title/Abstract])) OR (Prognostic marker[Title/Abstract])

Embase
A search with this search string was performed on 21-8-2023

('ANCA associated vasculitis')/br OR (('anti-neutrophil cytoplasmic autoantibody associated vasculit*'):ti,ab,kw) OR (('Anti-Neutrophil Cytoplasmic Antibody-Associated Vasculit*'):ti,ab,kw) OR (('Anti Neutrophil Cytoplasmic Antibody Associated Vasculit*'):ti,ab,kw) OR ((ANCA-vasculit*):ti,ab,kw) OR (('ANCA-Associated Vasculit*'):ti,ab,kw) OR (('ANCA Associated Vasculit*'):ti,ab,kw) OR ((GPA):ti,ab,kw) OR ((EGPA):ti,ab,kw) OR ((MPA):ti,ab,kw) OR (('granulomatosis with polyangiitis'):ti,ab,kw) OR (('eosinophilic granulomatosis with polyangiitis'):ti,ab,kw) OR (('microscopic polyangiitis'):ti,ab,kw) OR (('Churg Strauss'):ti,ab,kw) OR ((Churg-Strauss):ti,ab,kw) OR (('Eosinophilic Granulomatous Vasculit*'):ti,ab,kw) OR (('Granulomatosis with Polyangiitides'):ti,ab,kw) OR ((Wegener*):ti,ab,kw) OR (('Microscopic Polyangiitides'):ti,ab,kw) AND (CD163*)/br OR ((sCD163*):ti,ab,kw) OR ((usCD163*):ti,ab,kw) OR ((CD25*):ti,ab,kw) OR ((sCD25*):ti,ab,kw) OR ((usCD25*):ti,ab,kw) OR (('Interleukin 2 Receptor*'):ti,ab,kw) OR (('Interleukin-2 Receptor*'):ti,ab,kw) OR ((Interleukin-2R*):ti,ab,kw) OR (('Interleukin 2R*'):ti,ab,kw) OR ((IL-2R*):ti,ab,kw) OR (('IL 2R*'):ti,ab,kw) OR ((CD206*):ti,ab,kw) OR ((sCD206*):ti,ab,kw) OR ((usCD206*):ti,ab,kw) OR (('Mannose receptor'):ti,ab,kw) OR (('Mannose Receptor 1'):ti,ab,kw) OR (('Mannose Receptor C-Type I'):ti,ab,kw) OR (('MRC1 protein'):ti,ab,kw) OR ((sMCP-1):ti,ab,kw) OR ((CCL2):ti,ab,kw) OR (('Monocyte Chemotactic' and 'Activating Factor'):ti,ab,kw) OR (('Monocyte Chemoattractant Protein-1'):ti,ab,kw) OR (('Monocyte Chemoattractant Protein 1'):ti,ab,kw) OR ((Chemokine ( 'C-C Motif' ) 'Ligand 2'):ti,ab,kw) OR (('Monocyte Chemotactic Protein-1'):ti,ab,kw) OR (('Monocyte Chemotactic Protein 1'):ti,ab,kw) OR ((MCP-1):ti,ab,kw) OR (('MCP 1'):ti,ab,kw)

Cochrane

A search with this search string was performed on 18-08-2023

#1 (CD163):ti,ab,kw OR (sCD163):ti,ab,kw OR (usCD163):ti,ab,kw OR (CD25):ti,ab,kw OR (sCD25):ti,ab,kw (Word variations have been searched)

#2 (usCD25):ti,ab,kw OR (Interleukin 2 Receptor):ti,ab,kw OR (Interleukin-2 Receptor):ti,ab,kw OR ("IL-2 R"):ti,ab,kw OR ("IL-2 R alpha"):ti,ab,kw (Word variations have been searched)

#3 ("IL-2 R alpha chain"):ti,ab,kw OR ("IL-2 R alpha chains"):ti,ab,kw OR ("IL-2 R α"):ti,ab,kw OR ("IL-2 R α chain"):ti,ab,kw OR ("IL-2 R α chains"):ti,ab,kw (Word variations have been searched)

#4 ("IL-2 binding site"):ti,ab,kw OR ("IL-2 binding sites"):ti,ab,kw OR ("CD-25"):ti,ab,kw OR (Interleukin-2R):ti,ab,kw OR (Interleukin 2R):ti,ab,kw (Word variations have been searched)

#5 (IL 2R):ti,ab,kw OR (CD206):ti,ab,kw OR (sCD206):ti,ab,kw OR (usCD206):ti,ab,kw OR (mannose receptor):ti,ab,kw (Word variations have been searched)

#6 (Mannose Receptor 1):ti,ab,kw OR (Mannose Receptor C-Type I):ti,ab,kw OR (MRC1 protein):ti,ab,kw OR (sMCP-1):ti,ab,kw OR ("MCP-1"):ti,ab,kw (Word variations have been searched)

#7 ("MCP 1"):ti,ab,kw OR ("CCL-2"):ti,ab,kw OR ("CCL2"):ti,ab,kw OR (Monocyte Chemotactic and Activating Factor):ti,ab,kw OR ("monocyte chemoattractant protein 1"):ti,ab,kw (Word variations have been searched)

#8 ("monocyte chemoattractant protein-1"):ti,ab,kw OR ("monocyte chemotactic protein 1"):ti,ab,kw OR ("monocyte chemotactic protein-1"):ti,ab,kw OR ("CC chemokine ligand 2"):ti,ab,kw OR ("CC chemokine ligand-2"):ti,ab,kw (Word variations have been searched)

#9 (C-C):ti,ab,kw OR (Chemokine (C-C Motif) Ligand 2):ti,ab,kw OR ("MCP 1"):ti,ab,kw OR ("MCP-1"):ti,ab,kw (Word variations have been searched)

#10 ("ANCA associated vasculitides"):ti,ab,kw OR ("ANCA associated vasculitis"):ti,ab,kw OR ("ANCA-associated vasculitides"):ti,ab,kw OR ("ANCA-associated vasculitis"):ti,ab,kw OR (anti-neutrophil cytoplasmic autoantibody associated vasculits):ti,ab,kw (Word variations have been searched)

#11 ("Wegener granulomatosis"):ti,ab,kw OR ("Wegener's granulomatoses"):ti,ab,kw OR ("Wegener's granulomatosis"):ti,ab,kw OR ("Wegener's syndrome"):ti,ab,kw OR (Microscopic Polyangiitides):ti,ab,kw (Word variations have been searched)

#12 (Eosinophilic Granulomatous Vasculitis):ti,ab,kw OR (Eosinophilic Granulomatous Vasculitides):ti,ab,kw OR (Granulomatosis with Polyangiitides):ti,ab,kw OR ("Wegener granulomatoses"):ti,ab,kw OR ("Wegener"):ti,ab,kw (Word variations have been searched)

#13 (microscopic polyangiitis):ti,ab,kw OR ("Churg Strauss syndrome"):ti,ab,kw OR ("Churg-Strauss"):ti,ab,kw OR ("Churg-Strauss syndrome"):ti,ab,kw OR (Churg Strauss):ti,ab,kw (Word variations have been searched)

#14 (GPA):ti,ab,kw OR (EGPA):ti,ab,kw OR (MPA):ti,ab,kw OR (granulomatosis with polyangiitis):ti,ab,kw OR (eosinophilic granulomatosis with polyangiitis):ti,ab,kw (Word variations have been searched)

#15 #1 OR #2 OR #3 OR #4 OR #5 OR #6 OR #7 OR #8 OR #9

#16 #10 OR #11 OR #12 OR #13 OR #14

#17 #15 AND #16

**Supplementary Table S2**

*Biomarker kits*

| **Biomarker** | **Used biomarker kits/supplier** |
| --- | --- |
| usCD163/sCD163 | Human sCD163 DuoSet, DY1607; R&D Systems, Minneapolis, MN, USA  Quantikine® Human CD163 immunoassay, Bio-Techne R&D Systems |
| sCD206 | Human soluble Mannose Receptor HK381/CD206; HycultBiotech, Uden, The Netherlands |
| sCD25/usCD25 | Human sCD25; DY223; R&D Systems, Minneapolis, MN, USA  Human sIL-2R DY202, R&D Systems, Minneapolis, MN, USA  sIL-2R ELISA (DPC, Los Angeles, CA).  sIL2R (R&D Systems, Oxon, UK)  sIL2-R (T-cell Sciences, Cambridge. MA) |
| uMCP-1/sMCP-1 | Duoset DY279; R&D Systems, Minneapolis, MN, USA  R&D Systems, Oxon, UK  R&D systems, Abingdon, UK  Human Cytokine/Chemokine plex kit (Millipore, St. Charles, MO) on a multiplex platform (Eve Technologies Corp., Calgary, Alberta, Canada)  BD Biosciences |
